# *Oqtans*: a multifunctional workbench for RNA-seq data analysis

**DOI:** 10.1186/1471-2105-15-S3-A7

**Published:** 2014-02-11

**Authors:** Vipin T Sreedharan, Sebastian J Schultheiss, Géraldine Jean, André Kahles, Regina Bohnert, Philipp Drewe, Pramod Mudrakarta, Nico Görnitz, Georg Zeller, Gunnar Rätsch

**Affiliations:** 1 Computational Biology Center, Memorial Sloan-Kettering Cancer Center, New York, NY, USA; 2 Friedrich Miescher Laboratory, Max Planck Society, Tübingen, Germany; 3 Machine Learning/Intelligent Data Analysis Group, Technical University Berlin, Germany; 4 Structural and Computational Biology Unit, European Molecular Biology Laboratory, Heidelberg, Germany

## Background

The current revolution in sequencing technologies allows us to obtain a much more detailed picture of transcriptomes via deep RNA Sequencing (RNA-Seq). In considering the full complement of RNA transcripts that comprise the transcriptome, two important analytical questions emerge: what is the abundance of RNA transcripts and which genes or transcripts are differentially expressed. In parallel with developing sequencing technologies, data analysis software is also constantly updated to improve accuracy and sensitivity while minimizing run times. The abundance of software programs, however, can be prohibitive and confusing for researchers evaluating RNA-Seq analysis pipelines.

## Results

We present an open-source workbench, *Oqtans*, that can be integrated into the Galaxy framework that enables researchers to set up a computational pipeline for quantitative transcriptome analysis. Its distinguishing features include a modular pipeline architecture, which facilitates comparative assessment of tool and data quality. Within *Oqtans*, the *Galaxy*’s workflow architecture enables direct comparison of several tools. Furthermore, it is straightforward to compare the performance of different programs and parameter settings on the same data and choose the best suited for the task. *Oqtans* analysis pipelines are easy to set up, modify, and (re-)use without significant computational skill.

*Oqtans* integrates more than twenty sophisticated tools that perform very well compared to the state-of-the-art for transcript identification, quantification and differential expression analysis. The toolsuite contains several tools developed in the Rätsch Laboratory, but the majority of the tools were developed by other groups. In particular, we provide tools for read alignment (bwa, STAR, TopHat, PALMapper, …), transcript prediction (cufflinks, Trinity, Scripture, …) and quantitative analyses (DESeq2, edgeR, rDiff, rQuant, …). In addition, we provide tools for alignment filtering (RNA-geeq toolbox), GFF file processing (GFF toolbox) and tools for predictive sequence analysis (EasySVM, ASP, ARTS, …). See http://oqtans.org/tools for more details on included tools.

## Conclusions

*Oqtans* is integrated into the publicly available *Galaxy* server http://galaxy.cbio.mskcc.org which is maintained by the Rätsch Laboratory. It is also available as source code in a public GitHub repository http://bioweb.me/oqtans/git and as a machine image (managed by Galaxy CloudMan) for the Amazon Web Service cloud environment (instructions available at http://oqtans.org). *Oqtans* sets a new standard in terms of reproducibility and builds upon Galaxy’s features to facilitate persistent storage, exchange and documentation of intermediate results and analysis workflows.

Support: support@oqtans.org

Contact: vipin@cbio.mskcc.org

Details: http://oqtans.org

Public Computing Server: http://galaxy.cbio.mskcc.org

oqtans Demo Server: http://cloud.oqtans.org

oqtans Amazon Machine Image: ami-65376a0c

License: GPL http://www.gnu.org/licenses/gpl.html

